# Genome Comparison Identifies Different *Bacillus* Species in a Bast Fibre-Retting Bacterial Consortium and Provides Insights into Pectin Degrading Genes

**DOI:** 10.1038/s41598-020-65228-1

**Published:** 2020-05-18

**Authors:** Subhojit Datta, Dipnarayan Saha, Lipi Chattopadhyay, Bijan Majumdar

**Affiliations:** 1 Biotechnology Unit, Division of Crop Improvement, ICAR – Central Research Institute for Jute and Allied Fibres, Barrackpore, West Bengal 700 120 India; 2 Division of Crop Production, ICAR – Central Research Institute for Jute and Allied Fibres, Barrackpore, West Bengal 700 120 India

**Keywords:** Applied microbiology, Comparative genomics, Bacterial genomics

## Abstract

Retting of bast fibres requires removal of pectin, hemicellulose and other non-cellulosic materials from plant stem tissues by a complex microbial community. A microbial retting consortium with high-efficiency pectinolytic bacterial strains is effective in reducing retting-time and enhancing fibre quality. We report comprehensive genomic analyses of three bacterial strains (PJRB 1, 2 and 3) of the consortium and resolve their taxonomic status, genomic features, variations, and pan-genome dynamics. The genome sizes of the strains are ~3.8 Mb with 3729 to 4002 protein-coding genes. Detailed annotations of the protein-coding genes revealed different carbohydrate-degrading CAZy classes viz. PL1, PL9, GH28, CE8, and CE12. Phylogeny and structural features of pectate lyase proteins of PJRB strains divulge their functional uniqueness and evolutionary convergence with closely related *Bacillus* strains. Genome-wide prediction of genomic variations revealed 12461 to 67381 SNPs, and notably many unique SNPs were localized within the important pectin metabolism genes. The variations in the pectate lyase genes possibly contribute to their specialized pectinolytic function during the retting process. These findings encompass a strong foundation for fundamental and evolutionary studies on this unique microbial degradation of decaying plant material with immense industrial significance. These have preponderant implications in plant biomass research and food industry, and also posit application in the reclamation of water pollution from plant materials.

## Introduction

The unique versatility, biodegradability, and affordability of bast fibre of jute (*Corchorus olitorius* L. and *C. capsularis* L.), has earned it the name ‘golden fibre’. A sizeable population of South Asian farmers depend on jute cultivation. The phloic bast fibres in jute and mesta (*Hibiscus sabdariffa* L. and *H. cannabinus* L.), originating from cambium tissues are characterized by thick cells consisting of ligno-cellulosic materials. The fibre bundles are bonded together with other non-fibrous tissues and woody core by pectinous substances and hemicelluloses^1^. Removal of these cementing biopolymers for separating the fibres holds the key to the quality and strength of the fibres, and thus has remained the focus of research for quite a long time. The process of separation and extraction of bast fibres from jute and mesta stem by removing pectin and other complex carbohydrates is called retting, which exploits the actions of microbial community present in the retting water to decompose plant tissues.

Microbial activities influence the quality of fibre in hemp, flax, ramie, kenaf and jute^2–5^. The stems, when steeped in retting water release pectins, hemicelluloses and free sugars, which in turn promotes microbial growth. Microbes having pectinolytic and xylanolytic properties but no cellulolytic activity are considered efficient retting microbes^6^. Pectin of jute and mesta is highly methyl esterified and its degradation requires the combined action of a group of pectin degrading enzymes, polygalacturonase and pectin/pectate lyase^7,8^.

Several researchers have made notable contribution to isolate and identify the microbes responsible for the retting of bast fibres, including jute and mesta^6,9–11^. Banik *et al*.^12^ reported that the combined effects of urea and pectinolytic mixed bacterial culture reduced the retting time without explaining the role of enzymes involved. A composition of four *Bacillus* strains having pectinolytic, xylan and cellulose degrading abilities were used by Das *et al*. for ribbon retting of jute^5^. Notwithstanding the importance of these initial efforts, none of these microbial strains were further promoted and commercialized. An effective microbial retting consortium (CRIJAF SONA) was commercialized and adopted on a large-scale among jute growers, to reduce retting-time and enhancing fibre quality^13,14^. Application of CRIJAF SONA consortium during retting reduced the retting duration of jute by 7 days, with improved fibre recovery and fibre quality *i.e*. colour, lustre, fibre strength (27.0–28.1 g/tex, fineness (2.7–2.8 tex) and fibre recovery by 13.8–15.24% over control. The three *Bacillus* strains in the consortia have high polygalacturonase (PG) (5.1–6.0 IU/ml), pectin lyase (PNL) (185.7–203.7 IU/ml), and xylanase (15–16.2 IU/ml) activity^13^. The organisms present in microbial consortium were primarily identified up to species-level by metabolic fingerprinting pattern using Biolog system and further by ribotyping of a 977 bp 16 S rDNA fragment^13^.

Further improvement of the efficacy of the retting consortium warrants comprehensive molecular characterization of these strains and precise identification of genes and enzymes involved in the retting process. Comprehensive genome-scale analyses permit the unambiguous establishment of species and strain typing^15,16^. Also, verification of the functional uniqueness of these bacterial strains requires annotation of the complete set of genes, genomic variations, and specific gene analyses.

We report here, reliable genome-level taxonomic resolution of PJRB strains in retting consortium, CRIJAF SONA. We further inquired into the functional uniqueness of these bacterial strains through specific gene analyses and genomic variations.

## Materials and Methods

### The microbial retting consortium

The microbial consortium- consisting of three bacterial isolates (PJRB1 – Acc. No. MTCC 5573, PJRB2 - MTCC 5574, and PJRB3 - MTCC 5575) with high polygalactouronase, pectin lyase, and xylanase activity^13,14^ were used in the present study. Gram staining of the PJRB strains was performed using the standard protocol and visualized using light microscope (Olympus VX43). The size, morphology and other features were visualized using a scanning electron microscope (SEM, Hitachi S-530). Briefly, the protocol for sample preparation is as follows: log-phase cultures were fixed overnight in 0.25% glutaraldehyde (in 50 mM Na-Phosphate, pH 7.2) at room temperature. The bacterial cells were subsequently dehydrated for 10 min each in different ethanol grades (30%, 50%, 70%, 80%, and 90%) followed by storage in absolute ethanol prior to preparation of SEM stub. Finally, the bacterial cells were coated with gold in a sputterer and observed under scanning electron microscope at an electron high tension (EHT) of 5 kV.

The assay for extracellular pectinase enzyme was performed by inoculating the strains on pectin agar plates ameliorated with 1% citrus pectin followed by precipitation of the undigested pectin by soaking in 2% cetyl trimethyl ammonium bromide (CTAB) solution. The zone of substrate hydrolysis was measured as a ratio of diameter of clear zone to the diameter of colony as ‘potency index’.

### Genomic DNA isolation, library preparation, and sequencing

*Bacillus* strains were allowed to grow overnight in Luria Bertani broth at 37 °C and 5 ml overnight cultures were centrifuged at 5000×*g* for 10 min at 4 °C. Genomic DNA was extracted using PureLink® Genomic DNA Kits (Invitrogen # K1820–01). The quality of the final DNA samples was evaluated by gel electrophoresis (1.5% agarose gel) and DNA concentration was measured in NanoDrop 2000с Spectrophotometer (Thermo Scientific, MA, USA). Three separate paired-end (PE) sequencing libraries were prepared with NEB Next Ultra DNA Library kit (NEB #E7370). Three individual PE libraries for each PJRB strains were sequenced from AgriGenome Labs Private Limited, Kerala, India using Illumina HiSeq. 2500 platform at 100 × coverage. The adapter sequences were trimmed using TrimmomaticPE-0.39 program^17^. The low quality reads (Phred quality score Q < 30) were filtered out and the unique reads were fetched using FastUniq^18^. Additionally, FastQ Screen v0.13.0^19^ was used to sample the *Bacillus* origin of reads as a quality control measure before assembly.

### ***De novo*assembly, contig ordering and scaffolding**

*De novo* assembly was initially performed using three different assembly methods, *viz*. SPAdes v.3.13.0^20^, ABySS v.2.1.5^21^ and Velvet v.1.2.10^22^. The default k-mer sizes were used for SPAdes assembly, whereas, a range of k-mers from 31 to 95 was used for Velvet and ABySS assemblies. Finally, the ABySS derived assemblies with kmer 95 were used in further downstream analyses. Scaffolding and gap filling of the ABySS-assembled contigs were performed using SSPACE v.3.0 16^23^ and GapFiller v1–10^24^. After genome comparison for taxonomic classification, the assemblies were finally ordered against complete genomes of the reference strains (NZ_CP010405.1 – *B. safensis* FO-36b, NZ_CP031880.1 – *B. velezensis* OSY-GA1, and NZ_CP024204.1 – *B. altitudinis* P10) using Progressive MAUVE^25^. The ordered scaffolded assemblies were considered as ‘draft’ genomes. Genome wide-identification of phage-like and insertion sequences were performed using a webserver PHAST (PHAge Search Tool: http://phast.wishartlab.com)^26^, and ISEscan^27^.

### Genome-based taxonomic classification

For genome-scale taxonomic analysis, the genome assemblies were searched at Microbial Genomes Atlas (MiGA) online server (http://www.microbial-genomes.org)^28^ that uses NCBI non-redundant prokaryotic genomes database, JSpeciesWS (http://jspecies.ribohost.com/jspeciesws)^29^, and TrueBac^TM^ ID system from the EzBioCloud server (https://www.ezbiocloud.net/contents/genome). The species identification carried out in TrueBac-ID was based on the comparison of average nucleotide sequence identity (ANI) between the genomes of PJRB and their type strains. For a reliable identification of the strains, the Type (Strain) Genome Server (TYGS)^30^ was employed.

### Gene prediction and annotations

All three draft PJRB genomes were annotated using the NCBI Prokaryotic Genome Annotation Pipeline (PGAP)^31^. The functional annotation of genes was also carried out using web-based Rapid Annotation Using Subsystem Technology (RAST) annotation server (http://rast.theseed.org/FIG/rast.cgi)^32^. Protein coding genes were scanned for their organization into operons using the web server Operon-mapper^33^. Functional annotation of genes in terms of Kyoto Encyclopedia of Genes and Genomes (KEGG) orthology assignments and predictions of KEGG pathways were carried out through KEGG Automatic Annotation Server (KAAS) server (https://www.genome.jp/kegg/kaas/)^34^ using bi-directional best hit (BBH) method in GhostZ. Similarly, the PJRB genomes were also scanned for cluster of orthologous groups (COGs) annotations using eggNOG-mapper v2 (http://eggnog-mapper.embl.de)^35,36^. Automated Carbohydrate-active enzyme ANnotation web server, (dbCAN meta server (http://bcb.unl.edu/dbCAN2/blast.php)^37^, was employed to annotate Carbohydrate-Active enZYmes (CAZy)^38^. Potential interaction among the pectin and xylan-degrading CAZys were carried out using protein-protein interaction database, STRING v.11^39^.

For phylogenetic analyses of pectate lyase proteins, a total of 48 PLs from bacteria, fungi, nematode, and land plants were retrieved from the NCBI protein database. Domains for polysaccharide lyase (PL) were identified using SUPERFAMILY 2 database (http://supfam.org/)^40^ before analyzing their phylogenetic evolutions in MEGA X by MUSCLE alignment, Whelan And Goldman model (WAG) amino acid substitution model (Gamma distributed rates among sites) and maximum likelihood tree reconstruction^41^. Protein structure homology models, enzyme active site, and consensus ligand docking residues of PJRB pectate lyases were predicted using COFACTOR and COACH (https://zhanglab.ccmb.med.umich.edu/COACH/)^42^.

### Comparative genome analyses

Pan-genome comparative analyses of the PJRB strains were carried out using ROARY pipeline^43^. For comparison, complete genomes of strains of each species were retrieved from the NCBI database (Table S1). Both PJRB1 and PJRB2 genomes were compared with genomes of thirty strains each of *B. safensis* and *B. velezensis*, respectively. PJRB3 genome was compared with genomes of twenty-seven strains of *B. altitudinis*. The comparison and annotation of orthologous gene clusters among PJRB and their closely related strains were carried out using OrthoVenn2 (https://orthovenn2.bioinfotoolkits.net/home)^44^. For genome synteny and collinearity analyses, online tools D-GENIES^45^ and C-Sibelia software^46^ were used and alignment of syntenic blocks were visualized in Circos^47^.

### SNP identification

For SNP and variant identification among the bacterial strains, three separate tools *viz*. Snippy (https://github.com/tseemann/snippy), BactSNP (https://github.com/IEkAdN/BactSNP)^48^ and Parsnp-Gingr of Harvest suite^49^ were used. For Snippy and BactSNP, fastq raw reads of PJRB1, PJRB2, and PJRB3 were aligned against complete genomes of respective reference strains (NZ_CP010405.1, NZ_CP031880.1, and NZ_CP024204.1). In case of Parsnp, the PJRB1 and PJRB2 genomes were aligned to thirty genomes each of *B. safensis* and *B. velezensis*, respectively and PJRB3 genome with twenty-seven *B. altitudinis* genomes.

## Results

### Microscopy and pectinase activity of jute retting bacterial consortium

**Figure 1:**
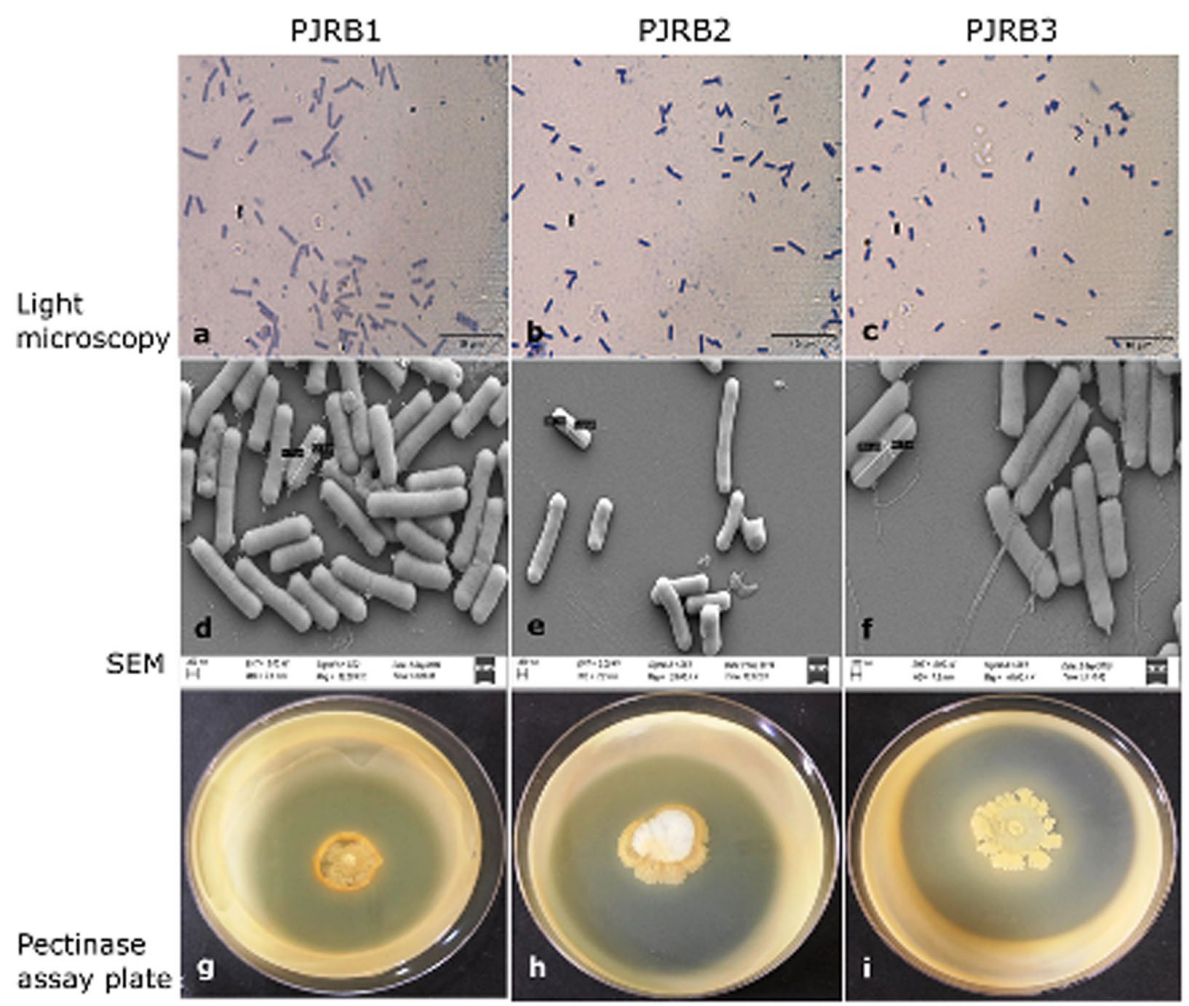
Morphological and phenotypic characterization of PJRB strains. Rod-shaped Gram positive bacterial cells with size are resolved in the upper and middle panel through light microscopy (**a–c**) and scanning electron microscopy (SEM) (**d–f**), respectively. Scale bars are shown in each figure. In the lower panel, the strains grown on pectin agar plates with 1% citrus pectin. Halo indicates the zone of substrate hydrolysis by the pectinolytic activity of PJRB1 (**g**), PJRB2 (**h**) and PJRB3 (**i**).

After Gram staining, the bacterial strains were observed as purple, rod-shaped cells under the compound light microscope indicating those are Gram-positive Bacilli (Fig. 1a–c). In order to examine macro structural features and differences on the surface of jute retting PJRB strains, we performed high-resolution SEM imaging (Fig. 1d–f). The micrograph showed that all three strains are rod shaped and have comparable shapes and size (length ~1.5 µM, width ~0.5 µM). The pectinase assay of the above PJRB strains showed high pectinase activity measured as potency index; PJRB1–2.8, PJRB2–2.6, and PJRB3–2.45. On pectin agar plates, the hydrolysis of pectin was observed as halo zones surrounding the bacterial colony amidst opaque area with residual pectin (Fig. 1g–i). The zone of substrate hydrolysis was measured as a ratio of diameter of clear zone to the diameter of colony as ‘potency index’ and all the three strains confirmed the potency index of >3 as reported by Das *et al*.^13^.

### Genome assemblies and genomic features of PJRB strains

Whole genome shotgun sequencing of the three PJRB strains were performed individually to generate ~4.0 GB data each with nearly 100 × genome coverage. The PE sequencing (mean read length 150 bp) generated approximately 16.5 to 17.0 million reads with an average Phred quality score of 38.8. After quality filter (Q > 30), approximately 14.6 to 14.8 million reads were assembled using three different tools SPAdes, Velvet, and ABySS (Table 1). On preliminary examination, out of the three assembly methods, the ABySS generated assemblies with kmer 95 were found to have consistent statistics and genome sizes comparable to other *Bacillus* strains and therefore, selected for further downstream analysis. The primary assemblies of PJRB1, PJRB2, and PJRB3 consisted of 3813976, 3880712, and 3899490 base pairs with N50 values of 788506 bp, 528477 bp, and 205868 bp respectively (Table 1).

**Table 1:** Sequence metrics and features of PJRB genomes.

Features	PJRB1	PJRB2	PJRB3
No. of reads	14,843,867	14,851,391	14,639,509
**Primary assemblies**			
***SPAdes***			
Assembly length	4,902,156	6,892,657	5,825,773
N50	708,884	569,445	173,580
***Velvet***			
Assembly length	8,060,469	6,240,148	3,722,406
N50	561	625	671
***ABySS***			
Assembly length	3,813,976	3,880,712	3,899,490
N50	259,390	458,936	98,293
**Final assembly post scaffolding and contig reordering**			
Scaffold Nos.	21	21	46
Total bases	3,809,132	3,876,440	3,883,973
Maximum scaffold length	981,857	1,096,081	392,651
N50	788,506	528,477	205,868
GC %	41.42	46.55	40.88

**Figure 2:**
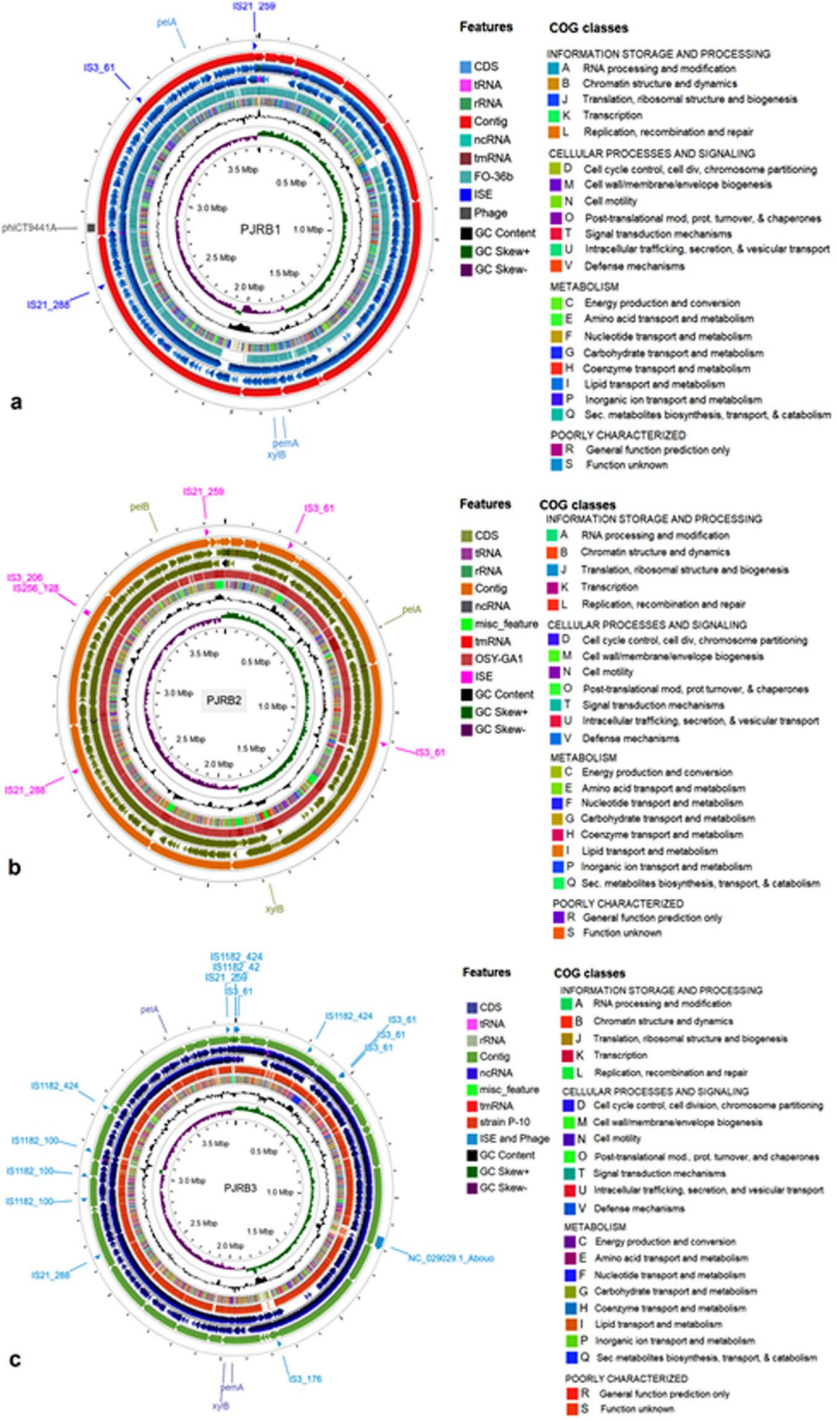
Circular representation of draft genomes and features of the PJRB strains. (**a**) Draft genome of PJRB1 aligned to *B. safensis* FO-36b strain; (**b**) PJRB2 aligned to *B. velezensis* OSY-GA1 strain; and (**c**) PJRB3 to *B. altitudinis* strain P-10. The contents of the featured rings (starting with the outermost ring to the centre) are as follows. Ring 1: Position of insertion (IS) and phage-like (phi) sequences; Ring 2: distribution of the scaffolds; Ring 3 and 4: ORFs in forward and reverse strands; Ring 5: BLASTn hits to reference *Bacillus* strains; Ring 6: Genome-wide COG class annotation of the PJRB genomes. Each COG classes are depicted in different colour; Ring 7: plots of GC content; Ring 8: GC skew plot, values above average is depicted in green and below average in purple. Important pectin degradation related genes were marked as *pel*A, *pel*B (Pectate lyase), *pem*A (Pectin esterase A) and *xyl*B (Xylulose kinase). The figures were produced using CGView Server^BETA^ (http://cgview.ca/).

In order to improve the genome assemblies, contigs were arranged to be part of larger scaffolds and ordered according to the complete genomes of respective closest strains (Fig. S1). The final scaffolded and reordered PJRB1, PJRB2, and PJRB3 assemblies consisted of 21, 21, and 46 scaffolds with a maximum size of 981857, 1096081 and 392651 bp, respectively. The final genome size of PJRB1, PJRB2, and PJRB3 consisted of 3809132, 3876440 and 3883973 base pairs with a GC percentage of 41.42, 46.55, and 40.88, respectively (Fig. 2). PJRB1 genome comprised three insertion elements and one intact phage sequence, PJRB2 genome comprised six insertion sequence and no intact phage, while the PJRB3 genome comprised 14 insertion sequences and one intact phage (Fig. 2).

### Genome-level resolution of taxonomic identities

Previously, with 16 S rDNA sequencing and metabolic fingerprinting in Biolog, the PJRB1, PJRB2, and PJRB3 were identified as strains of *Bacillus pumilus*^14^. Notwithstanding the importance of these methods in initial classification, the genome sequences of the strains are imperative for insights into their precise taxonomic status using different genome-based taxonomic tools. For PJRB1, the closest relatives found in the MiGA database were *B. safensis* CP032830 (98.85% ANI) and *B. safensis* NZ CP018197 (98.28% ANI). PJRB1 was suggested to most likely belong to the species *B. safensis* (p-value: 0.0016) or to the same subspecies of *B. safensis* CP032830 (p-value: 0.051). Similarly, the closest relative of PJRB2 was *B. velezensis* NZ CP031880 (99.51% ANI). This strain most likely belongs to either *B. velezensis* (p-value: 0.0008) or the same subspecies of *B. velezensis* NZ CP031880 (p-value: 0.05). In case of PJRB3, the closest relatives found by MiGA was *B. altitudinis* NZ CP024204 (98.23% ANI). PJRB3 therefore, most likely belongs to the species *B. altitudinis* (p-value: 0.0032) or to the same subspecies of *B. altitudinis* NZ CP024204 (p-value: 0.053). The RDP classifier which estimates the broad taxonomy of bacterial cells using 16 S rRNA training set, classified all the above three strains as *Bacillus* spp. with 100% confidence.

The taxonomic identities of the above three strains were further confirmed using the tools TrueBac ID from the EzBioCloud server and TYGS server. Based on genomic evidence, the above tools confirmed the identity of these strains as PJRB1: *B. safensis* (97.5% genomic similarity and ANI 97.5%); PJRB2: *B. velezensis* (97.8% genomic similarity and ANI 97.8%), and PJRB3 as *B. altitudinis* (98.3% genomic similarity and ANI 98.3%). Using Genome BLAST Distance Phylogeny (GBDP) approach of TYGS, the three query strains were assigned to 14 species clusters and were grouped accordingly with the respective species (Fig. S2).

### Pan-genome comparative analyses of PJRB strains and genome synteny

In order to compare the functional genes in terms of core and accessory genes shared or unique among strains, the PJRB strains in their respective clades were inferred at pan genome-scale (Fig. S3). The PJRB1 genome was analyzed based on 9363 gene clusters of thirty-one *B. safensis* strains. Out of 9363 orthologous gene clusters, 2727 genes (29.13%) constituted the core-genome (present in >99% of genomes), and only 214 (2.29%) as soft core genes (in 95–99% of genomes). Shell genes (present in 15–95% of genomes) and cloud genes (present in 0–15% of genomes) were 1370 and 5050, respectively indicating that 68.59% genes present as either shell genes or cloud genes. The pan-genome of PJRB2 and other *B. velezensis* strains comprised a total of 8107 genes as gene cluster, with 37.18% as core genes and 60.59% as shell and cloud genes. Although the *B. altitudinis* pan-genome was comprised of least number of 7751 gene cluster, the proportion of core genes was highest (38.45%) and shell and cloud genes together comprised slightly less than 60% of the pan-genome. The above analysis indicates a considerable proportion of genes comprised the unshared portion in shell and cloud genes. Moreover, for all the above three pan genomic analyses the size of pan-genome increased with further addition of genomes (Fig. S4).

**Figure 3:**
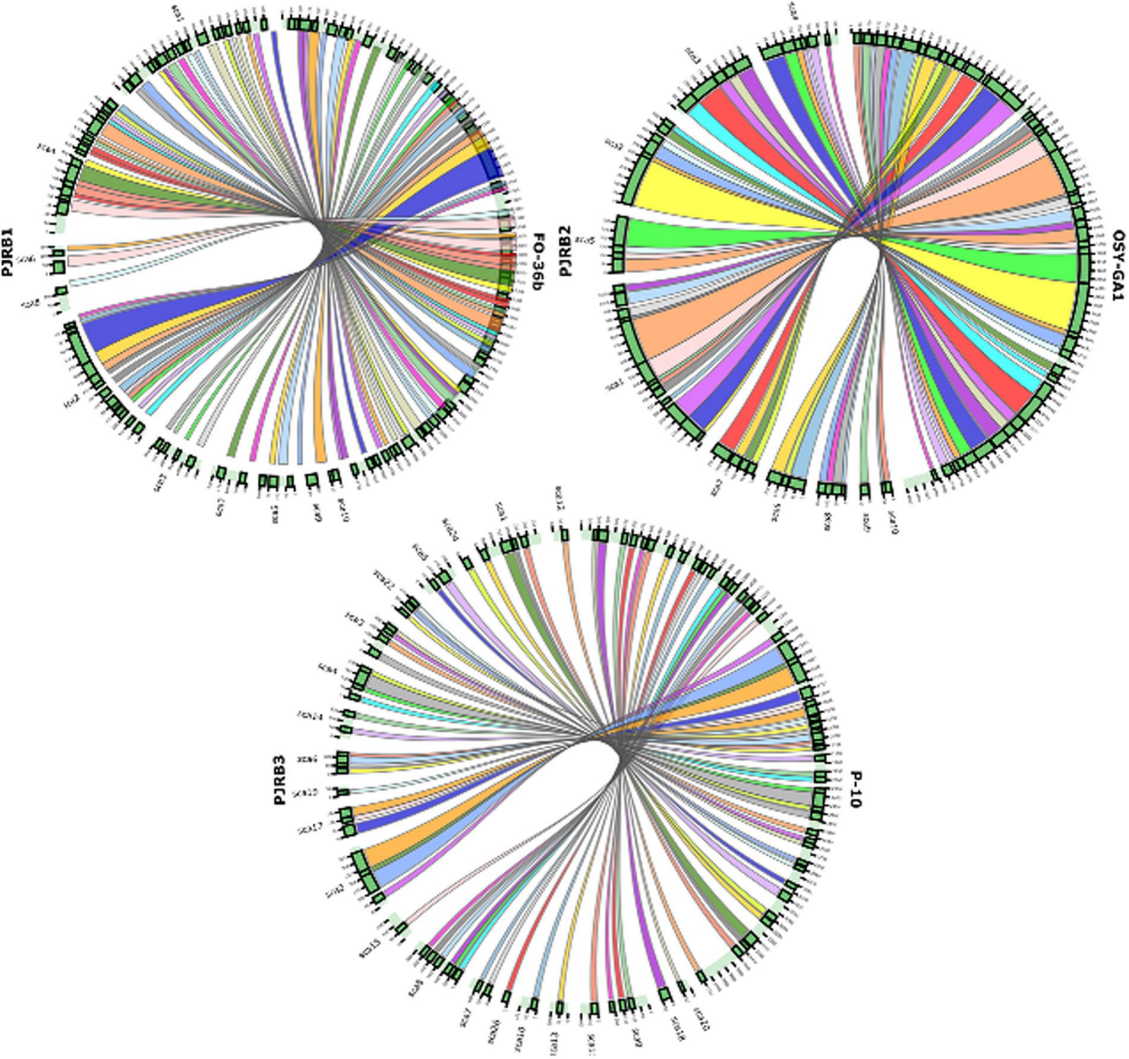
Genomic synteny shared between PJRB and their phylogenetically nearest strains. The pairwise multiple synteny blocks between PJRB1 and FO-36b strains showed 58 syntenic regions distributed in 10 scaffolds. Similarly, PJRB2 and OSY-GA1 strains showed 48 syntenic blocks distributed in 10 scaffolds and PJRB3 and P-10 showed 51 syntenic blocks distributed in 22 scaffolds. The syntenic diagrams were generated using Circos as an inbuilt tool of C-Sibelia^46^.

Pair-wise genome alignments of PJRB strains with other strains from same species revealed that they have very high genomic similarity and collinearity. No significant genomic rearrangements were evident from the dot plots except few chromosomal deletions. Chromosomal inversion was observed only in case of *B. altitudinis* strains PJRB3 and GR8. The pairwise comparison of multiple alignment blocks between PJRB1 and FO-36b strains showed 58 syntenic regions distributed in 10 scaffolds. Similarly, PJRB2 and OSY-GA1 strains showed 48 synteny blocks distributed in 10 scaffolds and PJRB3 and P-10 showed 51 synteny blocks distributed in 22 scaffolds (Fig. 3).

### Genome annotation of jute retting bacterial consortium

The genome assemblies of PJRB1, PJRB2, and PJRB3 were annotated in-depth using the NCBI PGAP (Table 2; details in Tables S2–S4). The genome of PJRB3 contains highest number of 3901 genes organized in 2158 operons and PJRB2 had 3699 protein coding genes in 2008 operons; whereas PJRB1 had 3780 protein coding genes in 2034 operons. In contrast, PJRB3 consisted lowest number of RNA coding genes (88 genes) compared to PJRB2 and PJRB1 (111 and 89 RNA coding genes, respectively). Pseudogenes found in these three assembles were 47, 99, and 88, respectively for the PJRB1, PJRB2, and PJRB3 genomes.

**Table 2:** Annotation details of PJRB genomes through NCBI Prokaryotic Genome Annotation Pipeline (PGAP).

Features	PJRB1	PJRB2	PJRB3
Total genes	3,916	3,909	4,077
CDSs	3,827	3,798	3,989
Proteins	3,780	3,699	3,901
Total RNA	89	111	88
rRNAs			
5 S	3	7	5
16 S	3	4	2
23 S	4	5	1
tRNAs	74	90	75
ncRNAs	5	5	5
Pseudo genes	47	99	88

The functional annotation tool RASTtk, which provides accurate subsystem level annotations besides predicting number of genes, was also employed to analyse the PJRB1, PJRB2, and PJRB3 genomes. The number of genes (including both protein encoding and RNA genes) were 4078, 4054, and 4258, respectively for the three genomes. As per RASTtk seed subsystem annotations, 1271 genes (32%) of PJRB1 fall into 337 subsystems, 1246 genes (32%) of PJRB2 into 342, and 1267 genes (31%) of PJRB3 into 345 subsystems. Among different subsystem categories ‘amino acid and derivatives’ comprised highest number of genes (350 in PJRB1, 321 in PJRB2, and 328 in PJRB3) followed by ‘carbohydrates’ (267 in PJRB1, 234 in PJRB2, and 276 in PJRB3) (Fig. S5).

### Analysis of orthologous genes in PJRB strains

**Figure 4:**
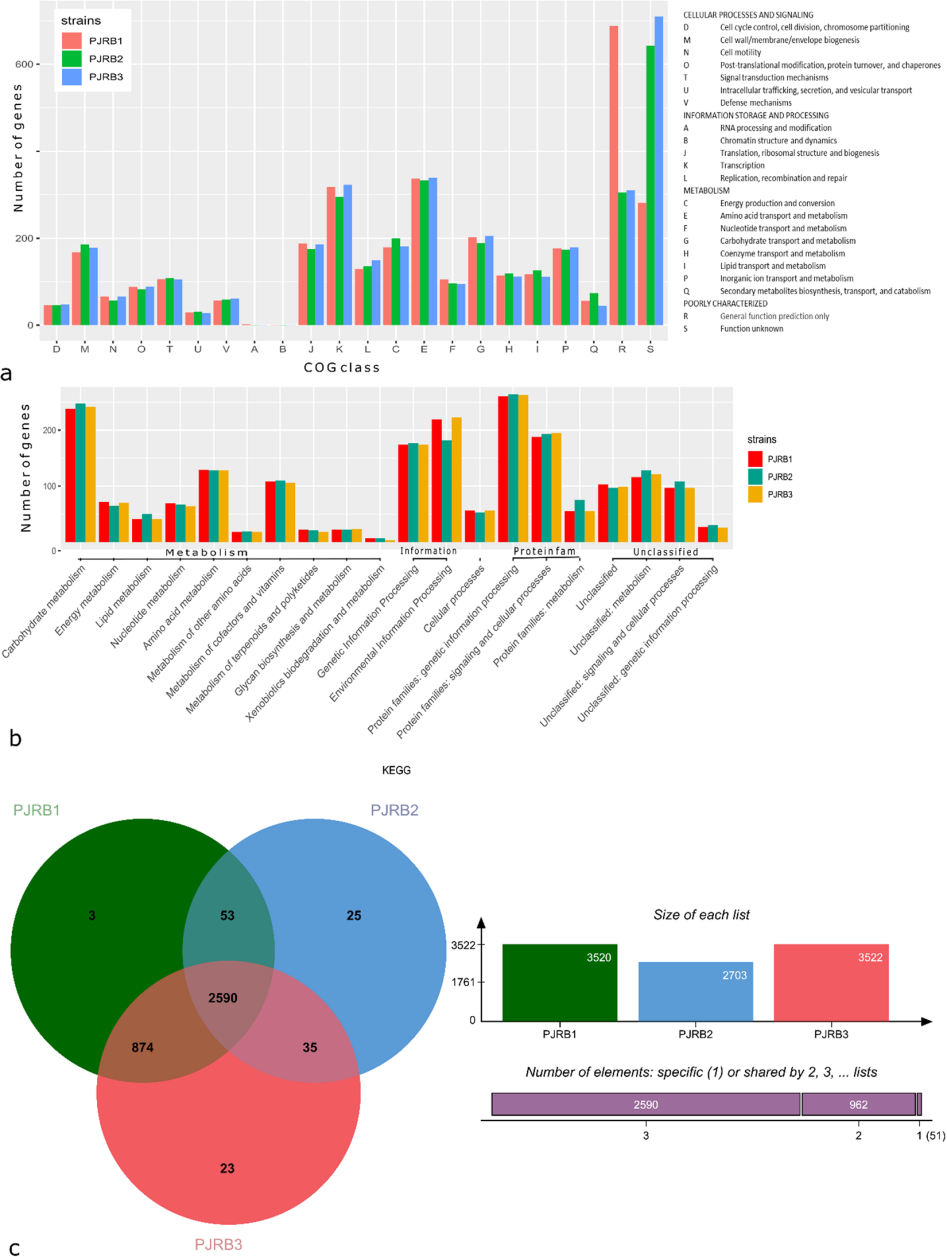
Analysis of orthologous genes in PJRB strains using COG, KEGG, and OrthoVenn. (**a**) Bar plot showing number of genes under 22 different COG categories depicted on X-axis according to four broad functional groups. (**b**) KEGG pathway enrichment analysis represented through bar chart shows distribution of number of proteins in 20 different KEGG functional categories annotated using the KAAS. (**c**) Venn diagram represents distribution of shared and unique gene clusters among different PJRB strains.

Clusters of orthologous groups (COG) annotation of the PJRB genomes revealed that 88.87% to 91.32% of the protein-coding genes were annotated using COG database (Fig. 4a) (details in Tables S5–S7). Among the COG functions, a substantial fraction of genes (36.54% to 38.81%) was involved in ‘metabolism’; of which ‘amino acid transport and metabolism (E)’ is the predominant functional category (333 to 339 genes). The other COG functional categories consisted ‘information storage and processing’ of about 18% and ‘cellular process and signalling’ of about 16.5% of the COG categories. *viz*. cell wall/membrane/envelope biogenesis (M168–186 replication, recombination and repair; L129–149 signal transduction mechanisms; T 105–108 translation, ribosomal structure and biogenesis; J 175–186 transcription; K 295–318).

Furthermore, KEGG pathway enrichment analysis could annotate nearly half of the total protein-coding genes, majority of which are involved in metabolic processes (Fig. 4b). Carbohydrate metabolism (~240 genes) and amino acid metabolism (~128 genes) constitute the predominant categories of metabolic genes. In each of the PJRB strains ~22 genes are involved in ‘biosynthesis and metabolism of glycans’. Xenobiotics biodegradation and metabolism is another important category of genes in KEGG analysis. PJRB1 and PJRB2 genome contained seven genes under this category, whereas PJRB3 consisted only three such genes. Apart from the metabolism-related genes, protein families for genetic information processing (~262 genes) and protein families for signalling and cellular processes (~190 genes) also correspond to the major share of KEGG classifications in all the three PJRB genomes.

At the protein sequence level, analysis with OrthoVenn exhibited that the three strains form 3603 clusters, 1036 orthologous clusters (at least contains two species), and 2567 single-copy gene clusters (Fig. 4c). Number of ortholog clusters shared by all three species were 2590, while 962 clusters were shared by at least two genomes. A total of 51 gene clusters were specific to only a single genome. Out of the 51 gene clusters, three belonged to PJRB1, whereas, 25 and 23 specific gene clusters were from PJRB2 and PJRB3, respectively.

### Major CAZy categories and pectin degradation genes

Since PJRB strains are essentially selected for their pectin degradation characteristics, it is imperative to analyse the genome-wide distribution of genes encoding CAZys. Total number of CAZymes were highest in strains of *B. velezensis* (88–108) as compared to that of *B. safensis* (77–81) and *B. altitudinis* (79–83) (Table 3) (details in Tables S8–S10). The major carbohydrate degrading CAZy classes observed are PL1, PL9, GH28, CE8, and CE12. Among the CAZy classes, the glycoside hydrolase family 28 (GH28) was most predominant with their numbers varying between 32–39 among different strains of *B. safensis*, *B. velezensis* and *B. altitudinis*. Among the three strains, PJRB2 contained maximum 38 GHs, while PJRB1 and PJRB2 consisted 35 GHs each. With a closer look on the polysaccharide lyase CAZy family, which encodes pectin degrading enzymes like exo-pectate lyase (PL1; EC 4.2.2.9) and exopolygalacturonate lyase (PL9; EC 4.2.2.9), one copy each of PL1 and PL9 were observed in PJRB1 and PJRB3 strains. Whereas, PJRB2 genome consisted two copies of PL1 and single PL9. Carbohydrate esterase family 8 (CE8) encoding for pectin methylesterase (EC 3.1.1.11) and CE12 for pectin acetylesterase (EC 3.1.1.6), which catalyze the acylation of carbohydrates constitutes an important CAZy family. The number of CEs varied between 13–15 in *B. altitudinis* strains, while all strains of *B. safensis* contained exactly 15 CEs. Although strains of *B. velezensis* contained fewer CEs (11), PJRB2 in contrast contained 20 CEs. By incorporating the STRING interaction results with the pectin and xylan degrading enzymes of other *Bacillus* species, we could predict an enzyme interaction network based on co-occurrence of the respective genes across the genomes (Fig. S6). Pectin esterase, derived by automated computational analysis using gene prediction method, was found to interact with arabinoxylan hydrolase. Pectin lyase, catalyzing the depolymerization of methyl-esterified pectins was found to interact with rhamnogalacturonan acetylesterase, which play a considerable role in the degradation of rhamnogalacturonan derived from plant cell walls. Similarly, pectate lyase was found to interact with glycoside hydrolases (Table S11).

**Table 3:** Genome-wide comparative distribution of CAZymes in PJRB genomes and selected *Bacillus* species.

Bacterial species and strains	Total CAZy*	Major CAZy categories						Pectinase coding CAZys
		GT	GH	PL	CE	AA	CBMs	
**B. safensis PJRB1**	78	25	35	2	15	0	3	PL1, PL9, GH28,CE8, CE12
B. safensis FO-36b	75	24	33	2	15	0	3	PL1, PL9, GH28, CE8, CE12
B. safensis U14-5	76	23	35	2	15	0	3	PL1, PL9, GH28, CE8, CE12
B. safensis BRM1	75	24	33	2	15	0	3	PL1, PL9, GH28, CE8, CE12
B. safensis U41	77	25	33	2	15	0	4	PL1, PL9, GH28, CE8, CE12
B. safensis KCTC 12796BP	79	24	36	2	15	0	4	PL1, PL9, GH28, CE8, CE12
**B. velezensis PJRB2**	84	36	38	3	20	5	6	PL1 (2), PL9
B. velezensis OSY-GA1	85	32	37	3	11	1	4	PL1 (2), PL9
B. velezensis SQR9	87	32	39	3	11	1	4	PL1 (2), PL9
B. velezensis L-S60	85	32	37	3	11	1	4	PL1 (2), PL9
B. velezensis JS25R	88	33	39	3	11	1	5	PL1 (2), PL9
B. velezensis AS43.3	85	32	37	3	11	1	4	PL1 (2), PL9
**B. altitudinis PJRB3**	81	28	35	2	14	0	4	PL1, PL9, GH28, CE8, CE12
B. altitudinis P-10	78	27	34	2	13	0	4	PL1, PL9, GH28, CE8, CE12
B. altitudinis GR-8	78	27	33	2	14	0	4	PL1, PL9, GH28, CE8, CE12
B. altitudinis W3	80	27	34	2	15	0	4	PL1, PL9, GH28, CE8, CE12
B. altitudinis GQYP101	80	27	34	2	15	0	4	PL1, PL9, GH28, CE8, CE12
B. altitudinis HQ-51-BA	77	27	32	2	14	0	4	PL1, PL9, GH28, CE8, CE12

*Consensus CAZy predictions by HMMER, Diamond BLAST hits, and HotPep.

GHs - Glycoside hydrolases; GTs - Glycosyl transferases; PLs - Polysaccharide lyases; CEs - Carbohydrate esterases; AAs - Auxiliary activities; CBMs - Carbohydrate-binding modules.

### Phylogenetic analyses and homology modelling of pectate lyases

**Figure 5:**
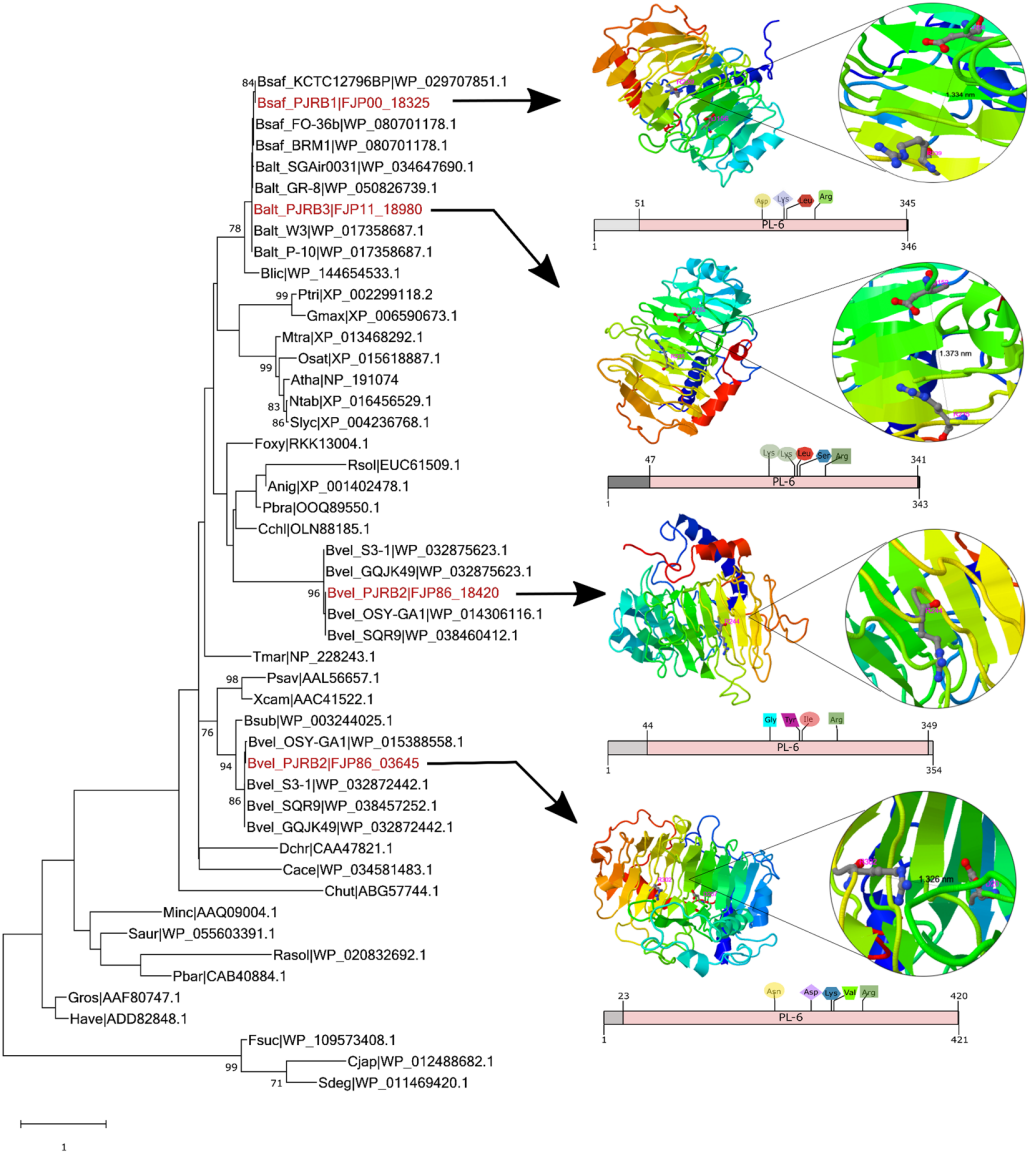
Phylogenetic relationship of pectate lyases in PJRB strains with other organisms. The Maximum Likelihood tree of 48 pectate lyase proteins were constructed using WAG amino acid substitution model with Gamma (G) distributed rates among sites and complete deletion of gaps and missing data. The tree with the highest log likelihood (−5121.95) is shown. PJRB strains are marked in red. Bsaf: *Bacillus safensis*; Bvel: *Bacillus velezensis*; Balt: *Bacillus altitudinis*; Bsub: *Bacillus subtilis*; Rasol: *Ralstonia solanacearum*; Blic: *Bacillus licheniformis*; Psav: *Pseudomonas savastanoi*; Xcam: *Xanthomonas campestris*; Saur: *Streptomyces aureus*; Dchr: *Dickeya chrysanthemi*; Tmar: *Thermotoga maritima*; Pbar: *PaeniBacillus barcinonensis*; Cace: *Clostridium acetobutylicum*; Cjap: *Cellvibrio japonicus*; Sdeg: *Saccharophagus degradans*; Fsuc: *Fibrobacter succinogenes*; Chut: *Cytophaga hutchinsonii*; Minc: *Meloidogyne incognita*; Gros: *Globodera rostochiensis*; Have: *Heterodera avenae*; Cchl: *Colletotrichum chlorophyti*; Foxy: *Fusarium oxysporum*; Rsol: *Rhizoctnia solani*; Pbra: *Penicillium brasilianum*; Anig: *Aspergillus niger*; Atha: *Arabidopsis thaliana*; Ntab: *Nicotiana tabacum*; Ptri: *Populus trichocarpa*; Slyc: *Solanum lycopersicum*; Mtra: *Medicago truncatula*; Gmax: *Glycine max*; Osat: *Oryza sativa* japonica. Representative models of the PJRB pectate lyases derived from closest PDB homologues using COFACTOR module of COACH server are show in the right side with the active site residues denoted in pink. The respective Polysaccharide lyase-6 (PL-6) domains as identified using SUPERFAMILY 2, and the ligand docking residues are illustrated below the models.

Phylogenetic relationship was analyzed among 48 pectate lyase proteins from PJRB and their related strains and also from other well characterized bacteria (Fig. 5). Pectate lyase being a ubiquitous enzyme, sequences from fungi, nematode, and land plants were also included to analyze the diversity. The Maximum Likelihood tree grouped together all the pectate lyases from *Bacillus safensis* and *Bacillus altitudinis* including those of PJRB1 and PJRB3 as per their species affiliations. Whereas the two isoforms of pectate lyases of PJRB2 formed two separate and distinct clusters with *B. velezensis* strains OSY-GA1, S3–1, SQR9 and GQJK49. None of the *Bacillus* pectate lyases clustered with those of plants, fungi or nematodes.

In addition to the phylogenetic analysis, three dimensional structures of pectate lyases were also compared. *Ab initio* model prediction of pectate lyases from PJRB strains were performed to explore the structural divergence and identification of ligand binding and active site residues (Fig. 5). As per COACH analyses, the closest structural homologue of PJRB1 and PJRB2 pectate lyases was a hexasaccharide I bound to *B. subtilis* pectate lyase (PDB model 2NZM), whereas, the pectate lyase from PJRB3 strain found its closest homologue in pectate Lyase C of *Dickeya chrysanthemi* mutant R218K (PDB model 2EWE). In case of PJRB1, the ligand ADA (α-D-galacturonic acid) interacted with four residues of the enzyme- Asp 186, Lys 210, Leu 213 and Arg 244. Similarly, the ligand binding residues of PJRB2 PL2 comprised of Gly 185, Tyr 209, Ile 212 and Arg 250. The pectate lyase enzymes of PJRB strains exhibited considerable divergence in their sequence as evidenced in the ligand binding site residues predicted by *ab initio* modelling. Pectate lyase of PJRB3 and PL1 of PJRB2 had five residues each in their ADA binding site. In PJRB2 PL1, the binding residues are Asn 203, Asp 246, Lys270, Val 273 and Arg 307; whereas in PJRB3 PL these residues are Lys 178, Lys 206, Leu 209, Ser 212 and Arg 240.

Active site residues were predicted by COFACTOR module of COACH, and, except PJRB PL2 which contained a single Arg at position 244, all other contained one Asp and one Arg each in their active sites. The closest active site homologue observed in PJRB1 and PJRB3 pectate lyases was a pectate lyase of *D. chrysanthemi* (PDB model 1pclA), whereas, both the PLs of PJRB2 had highest homology with that of *Bacillus* sp. TS-47 (PDB model 1vblA).

### Genome-wide variant identification in PJRB strains

Several genomic variants were discovered in PJRB genomes based on two different variant calling approaches by Snippy and BactSNP. The PJRB1 genome when compared to *B. safensis* strain FO-36b, PJRB2 to *B. velezensis* strain OSY-GA1, and PJRB3 to *B. altitudinis* strain P-10, they produced total sequence variants of 67381 (61001 in CDS), 12461 (11012 in CDS), and 41345 (37125 in CDS), respectively (Tables S12–S14). Majority of these variants (84%-90%) are SNPs (Table S15). Deletions are only around 0.5% in both PJRB1 and PJRB3, and slightly higher (1%) in PJRB2. An almost similar pattern was observed for insertion type variants. Other complex type variations are 5.8% in PJRB2 and nearly 12% in PJRB1 and PJRB3. However, only a fraction of these variations had any real effect on bacterial metabolism and growth, as majority of them were found to be of synonymous type mutations. Among the total variants in CDS, 47650 (78.11%) are categorized as synonymous variants without any change in the coded amino acids. Similarly, in PJRB2 and PJRB3, 71% and 77% variants are of synonymous types. The proportion of missense mutations (non-synonymous that effect codon change) are 21.44% in PJRB1, 28.18% in PJRB2, and 22.08% in PJRB3. Frameshift mutations which changes the reading frames of genes were rather rare (0.17% to 0.44%). Base substitutions are mainly transitions types (68.57% to 69.49%), and nearly 30% changes are due to transversions. Most frequent base substitution type was found G > A (17.44% to 18.28%); whereas, the least frequent was C > G (2.29% to 3.01%).

Based on genome alignment approach of phylogenetically close bacterial strains, 78301 SNPs in PJRB1, 13080 in PJRB2 and 48406 in PJRB3 were identified, out of which 2339, 909, and 3670 were found unique to PJRB1, PJRB2 and PJRB3, respectively. Interestingly, several SNPs were located in the pectin degradation related genes in all the PJRB genomes (Fig. S7a–c). A unique transition type SNP, T > C was located in gene (locus: FJP00_18325) coding for pectate lyase in the PJRB1 genome. Similarly, two SNPs, G > A and C > T in genes (loci: FJP86_03645 and FJP86_18420) coding for pectate lyases in PJRB2 genome, and an G > A in gene coding for pectin esterase A (locus: FJP11_10115) in PJRB3 genome were also identified. Although not yet validated, these unique SNPs in the genes coding for pectin degrading enzymes possibly could account for their high pectinolytic efficiency in the retting process.

## Discussion

Microbial retting of bast tissues from jute and other allied fibre crops, such as mesta, is crucial for extracting good quality fibres of economic importance. Bast fibres are secondary phloem tissues that are affixed to the inner bark and the outside of cambium tissues. The fibre extraction process involves water-borne microbial decomposition of stem tissues adhered together by complex substances including pectin, hemicellulose, xylan, and lignin^1^. Since bast fibres are cellulosic in nature, the retting microbes must not have cellulolytic activity but should possess pectin and xylan degrading enzymes^5,13^. The retting process involves the build-up of microbial communities by dissolution of sugars and nitrogenous substances from the plant stems. The aerobic *Bacillus* initiate the degradation of pectin and xylans till the anaerobic bacteria of possibly *Clostridium* genus replaces them because of diminished availability of oxygen in the retting environment^2^. Earlier we have isolated and characterized three individual pectinolytic and xylanolytic bacterial strains devoid of cellulolytic activity, characterized them as *Bacillus* sp., and applied them as commercial formulation for jute retting consortium named ‘CRIJAF SONA’^13,14^. This formulation has become very popular among the jute farmers owing to its ability to reduce retting time and improve fibre quality and fibre recovery, thereby increasing the net income of jute farmers.

Sustained commercial success of this bacterial consortium demands precise characterization of these strains at genome sequence level to identify the genes for further improvisation of their efficiency and molecular strain typing. To date, sequencing and comparative analysis of genomes of several *Bacillus* species have provided important insights about their evolution, genetics, and physiology including a wide range of applications in agriculture and industry. Here, we report whole-genome shotgun sequencing of the pectinolytic strains of three *Bacillus* sp. at sufficiently high genome coverage and typical genomic features including annotation and variations of the pectin-degrading genes.

### Genome assemblies and annotations of PJRB strains establish their genomic organization and typical features

The genome size and GC content are considered very important in order to understand the ability of any microorganism to adapt to varying environmental conditions^50^. Based on the available complete genomes of *Bacillus* at NCBI, it is evident that there is up to 79% of genome size variation (3.42 Mb to 6.13 Mb), exhibiting considerable plasticity in *Bacillus* species. Similarly, the GC content of the studied species also vary from 34.7% to 94.7%. Nearly 3.8 Mb genomes assembled in contigs of three PJRB strains of *Bacillus* in the present study are consistent with these genome sizes and GC content. The GC content of PJRB1 and PJRB3 differed with PJRB2, which had slightly higher GC content and these results are consistent with the average GC% of 46.3% of the selected *Bacillus* species. Gene content, number of tRNA sequences, and other features like phage-like sequences and insertion sequences were also found similar to the genomes of respective *Bacillus* species to which the PJRB strains belong. The insertion sequences in microbes are known to influence the genome plasticity and adaptability^51^. All these features, including the insertion sites observed in the PJRB genomes, may be crucial in analysing their influence in genome plasticity and adaptability to a complex environmental niche like jute-retting water.

### **Genome-level resolution of PJRB consortium reveal taxonomic affiliations to three*Bacillus*species**

One of the major highlights of the present genome sequence analysis of jute retting bacterial consortium is their definitive taxonomic resolution based on genome-based phylogeny. It is now widely accepted that whole-genome sequence-based taxonomy using core genome alignment and ANI values is a better approach than the conventional method of bacterial taxonomic studies and 16 S rRNA sequencing^52–54^. Prior to this genome analysis, identity of the strains of the consortium was established as *Bacillus pumilus* by morphological, biochemical, and 16 S rDNA sequencing^13^. However, different genome-based taxonomic analysis in this study unequivocally identified them as three different *Bacillus* species, *viz*. PJRB1-*Bacillus safensis*, PJRB2-*Bacillus velezensis*, and PJRB3-*Bacillus altitudinis*. Although ribotyping based on 16 S rRNA is widely used for bacterial classification, it is often inadequate for the species whose 16 S rRNA genes are highly conserved^55^. In case of *Bacillus cereus*, it has been demonstrated that whole genome sequence analysis provided higher precision over the 16 S rRNA gene sequence to resolve the taxonomic ambiguities^15^. Similarly, genome-based taxonomic analysis proved effective in the reclassification of *Paenibacillus riograndensis* as a genomovar of *P. sonchi*^56^. Our findings too lead us to revise the taxonomic resolution of the jute retting bacterial consortium strains into three different *Bacillus* species in contrary to their earlier classification as strains of *B. pumilus*^13^.

### Comparative **genomics of*Bacillus*strains highlights genome collinearity and gene orthology**

Pan-genome profiles of bacterial genomes shed light on their core and accessory genes with differences in genomic signatures. While the conserved core genes can efficiently identify bacterial genus-level identity from a microbiome, strain-specific accessory genes actually define lateral gene transfer with novel functions^57^. Therefore, a comprehensive analysis of pan-genome aids in understanding the essential and laterally transferred functions of a newly sequenced bacterial genome. Since we have taxonomically resolved the identity of jute retting bacterial consortium into three different bacterial species, we performed separate pan-genome analysis of PJRB strains. In all the analyses, the steady increase of pan-genome with each further addition of genome explains that they have large and open pan-genome. We found that for all the PJRB strains, ~30% of the protein-coding genes in pan-genome cluster were grouped as core and ~60% as accessory genes. These findings are in agreement with that of other bacterial species^58^. As expected, these core genes are mainly involved in basic metabolism function of bacteria like recombination and repair, membrane biogenesis, carbohydrate metabolism, etc., whereas the accessory genomic regions may be useful to infer evolutionary history and specific adaptation of the bacterial strains. A comparative analysis of predicted protein-coding genes for their orthologous relationship also revealed significant gene overlaps among the three PJRB genomes. The number of overlapped genes are very similar to the core pan-genome and also corroborated to high genomic synteny and collinearity. Though overlapped genes are considered least effective markers in bacterial phylogenomic analysis of closely related strains, they can be combined with locally collinear blocks for a robust phylogenomic inference^59^. Thus, the orthologous gene clusters identified from the pan-genome analysis will serve as a solid platform for an in-depth assessment of genome translocations, horizontal gene transfer, and gene losses in these PJRB strains.

### Analysis **of carbohydrate-degrading genes exhibit distinct patterns and variations associated with PJRB strains**

Although, the carbohydrate degrading enzymes are currently classified into 23 families in the CAZy database, they fall into two main groups^38^. Glycoside hydrolases (GHs) catalyze the hydrolysis of glycosidic bonds and polysaccharide lyases (PLs) cleave uronic acid-containing polysacharides via a β-elimination mechanism. On some substrates, carbohydrate esterases (CEs) have also been reported to catalyze acylation of substituted saccharides. Whole-genome or transcriptome analysis are now routinely used in many bacterial species to locate or study the expression of genes that are involved in polysaccharide degradation. Often genes encoding CAZymes are reported to be organized in gene clusters. The extensive studies in the last few years have offered a number of insights into the complex enzymatic pathway required to degrade pectin^60,61^. Since the key characteristics of the studied bacterial strains of retting consortium are essentially based on efficient pectin degradation, the presence and organization of CAZymes constitute an important part of our study. We observed different carbohydrate degrading CAZy classes *viz*. PL1, PL9, GH28, CE8, and CE12 in all the three PJRB genomes, although their numbers varied from other strains of *B. safensis, B. velezensis* and *B. altitudinis*. Pectin lyase activity (U/ml) varies among the selected strains: PJRB1 185.7, PJRB2 197.7, and PJRB3 203.7^13^; this may possibly be attributed to variation in CAZymes in the genomes. Total annotated CAZymes were highest (84) in PJRB2, which also contained an extra copy of PL gene. It is also noteworthy that the commercial consortium contains PJRB2 in twice the concentration of the other two strains. Computational predictions also established an intricate enzyme interaction network of pectin degrading genes. This will facilitate to build models to further improve the synergy of this consortium for efficient retting.

Pectate lyase domain-based phylogenetic analysis of PJRB and other bacterial strains resolved their evolutionary convergence among the Firmicutes. Similarly, a phylogenetic tree based on 121 pectate lyases clustered them according to the source organisms such as bacteria, fungi, plants, and nematodes^61^. In another study, a multiple sequence alignment of 48 pectin lyase protein sequence of different bacteria and fungi revealed the presence of conserved motifs as well as variable active sites, explained their catalytic variabilities^60^. In this study, identification of ligand binding and active site residues through homology modelling of pectate lyases from PJRB strains offer an opportunity to improve the efficiency by modulating these sites. Changes in N terminal region of PelA protein of *Bacillus* sp. BP-23 PelA were demonstrated to render different substrate specificity and unusual features as compared to PelA proteins in other *Bacillus* species^62^. The large number of SNPs identified in the PJRB strains of *Bacillus* spp. reported here are expected to serve as crucial resources towards molecular typing and inference of intra-species genetic polymorphism. Several SNPs were located in pectin degradation related genes of all the PJRB strains and this is perhaps one of the first attempts to associate functional variation with SNPs in CAZy genes.

## Conclusions

CRIJAF SONA microbial formulation has helped in shortening the retting duration and improvement in fibre quality, providing the much-required fillip to the jute sector allowing diversification of jute products to tap the world market. Now with the availability of the complete genome sequences of the retting bacterial strains, it will be possible to further improve this technique by cloning and functional validation of the pectin degrading genes. In this study, the presence and organization of different carbohydrate degrading CAZymes classes *viz*. PL1, PL9, GH28, CE8, and CE12 in all the three PJRB genomes were established. Definitive genome-level taxonomic resolution of PJRB consortium revealed taxonomic affiliations to the three *Bacillus* species.

Also, a comprehensive understanding of the diversity of microbial population will help in incorporating other strains into the consortium and protecting IPR of the indigenous microbiome. Further application of high throughput next-generation sequencing can generate genome level data from each retting environment indicating the microbial diversity and their correlation with fibre quality. Genome analysis of the retting microbes provides additional insights into the mechanisms of carbohydrate metabolism and could be relevant for improving the next generation of efficient consortium. The global market for natural fibres is continuously growing due to changing environmental legislations and regulations, and a deeper understanding of retting process is prerequisite to achieve sustainable natural fibre production system.

## Data availability

Genome sequences of PJRB1, PJRB2 and PJRB3 are accessible at NCBI under the following accession nos.: PJRB1 (BioSample Accession SAMN11964560, Genome Accession VFLO00000000, SRA accession SRR9317474); PJRB2 (BioSample Accession SAMN11964574, Genome Accession VFLN00000000, SRA accession SRR9317475) and PJRB3 (BioSample Accession SAMN11964575, Genome Accession VFLM00000000, SRA accession SRR9317473).

## Supplementary information

Supplementary information.

Supplementary information2.
