# Supplementary material for: Genome Comparison Identifies Different Bacillus Species in a Bast Fibre-Retting Bacterial Consortium and Provides Insights into Pectin Degrading Genes
Source: Sci Rep. 2020 May 18;10:8169. doi: 10.1038/s41598-020-65228-1 (PMC7235092; doi:10.1038/s41598-020-65228-1)
Supplement: Supplementary file 2 — Supplementary information2. [file 41598_2020_65228_MOESM2_ESM.pdf]

Title:

Genome Comparison Identifies Different *Bacillus* Species in a Bast Fibre-Retting Bacterial Consortium and Provides Insights into Pectin Degrading Genes

Subhojit Datta[1], Dipnarayan Saha[1], Lipi Chattopadhyay[2] and Bijan Majumdar[2]

[1]Biotechnology Unit, Division of Crop Improvement, ICAR – Central Research Institute for Jute and Allied Fibres, Barrackpore, West Bengal – 700 120, India

[2]Division of Crop Production, ICAR – Central Research Institute for Jute and Allied Fibres, Barrackpore, West Bengal – 700 120, India

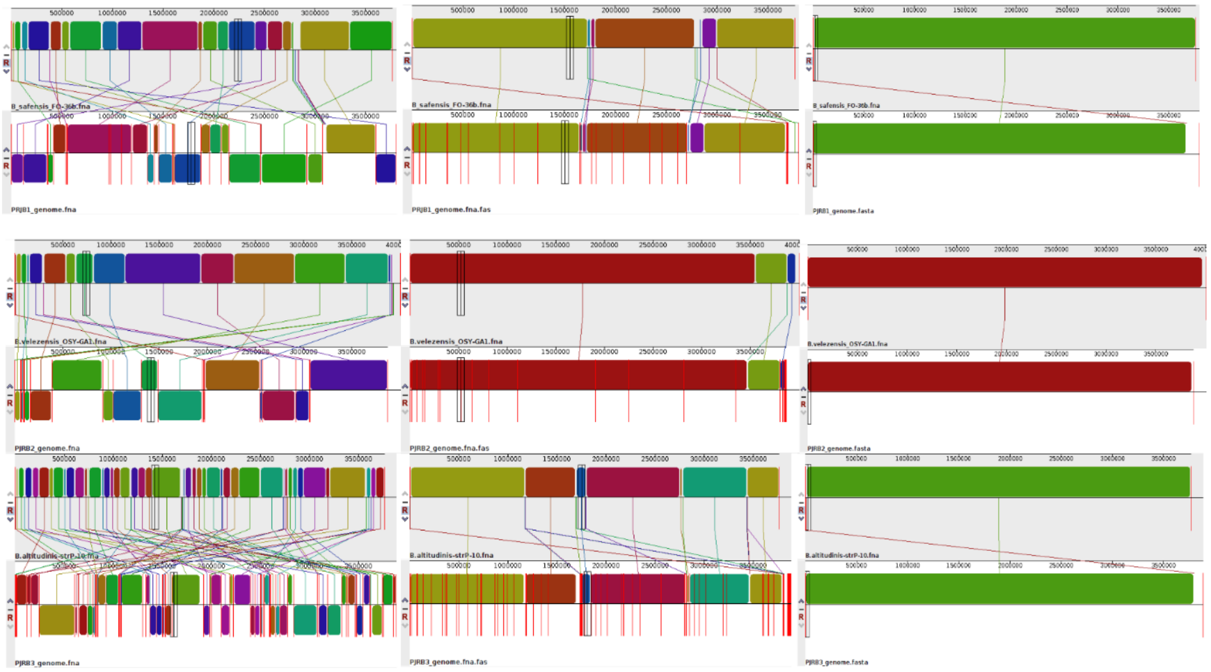

Fig. S1. Multiple genome alignment and reordering of scaffolds of PJRB strains was carried out using progressive Mauve. The left panel indicates alignment of scaffolds against the corresponding reference genomes. The reference complete genome used for PJRB1 was *B. safensis* strain FO-36b, for PJRB2 *B. velezensis* strain OSY-GA1, and for PJRB3 *B. altitudinis* strain P-10. Boxes with identical colours represent the local colinear blocks (LCB) and the LCBs below the black line have an inverse orientation relative to that of reference genome. The middle panel represent rearranged LCBs using the 'move contig' option of the progressive Mauve. The extreme right hand panel shows the final alignment of the draft genomes of PJRB strains to the complete genomes of reference strains.

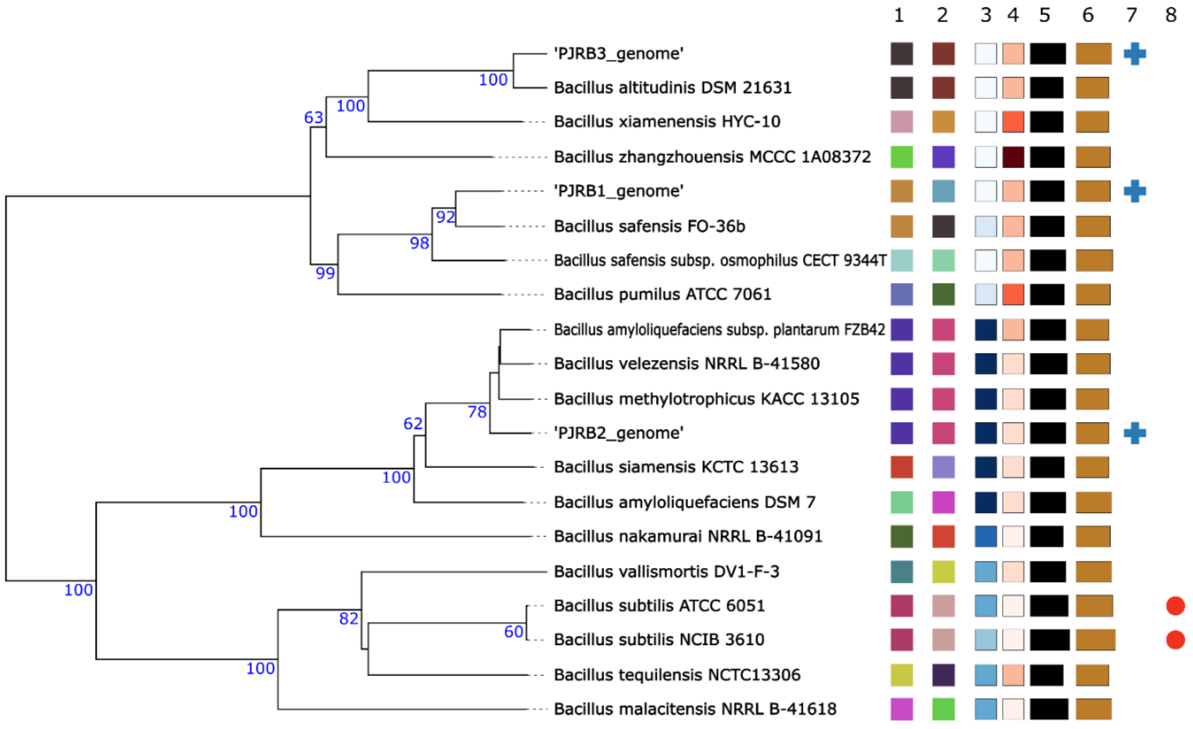

1: Species clusters; 2: Subspecies clusters; 3: Percent G+C (40.9-46.5%); 4: delta statistics (0.0-0.2)
5: Genome size (3611918-4275791 bp); 6: Protein count (3622-4345); 7: User strain; 8: Type species

Fig. S2.Genome based phylogeny of PJRB strains obtained from Type (Strain) Genome Server (TYGS). Whole-genome phylogenomic tree of PJRB and other *Bacillus* strains using Genome BLAST Distance Phylogeny (GBDP) approach of TYGS. Three query strains were assigned and grouped to different species clusters. Branch lengths are denoted in scales of GBDP distance calculation and numbers above branches are represent GBDP pseudo-bootstrap support values from 100 replications.

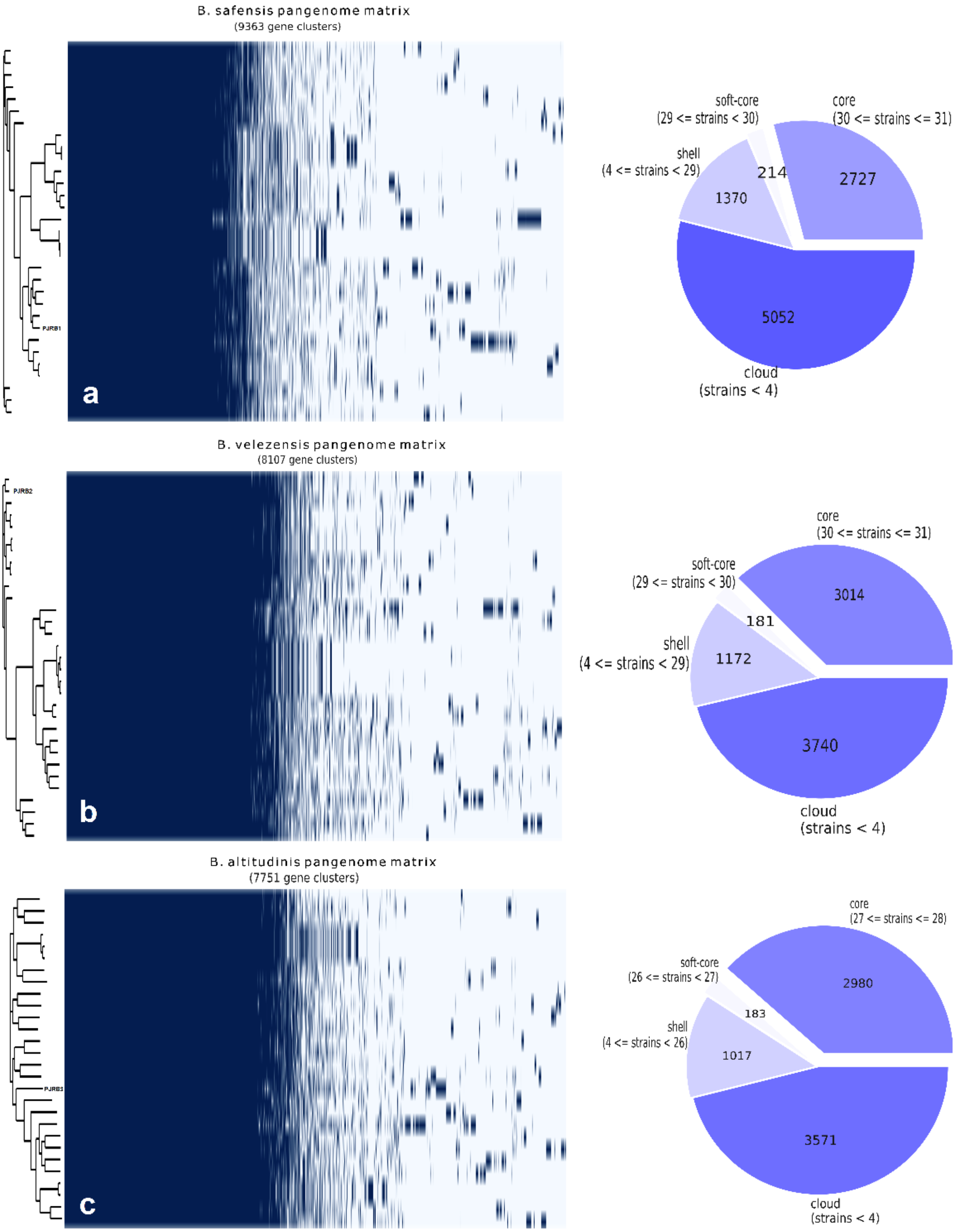

Fig. S3. Comparative pan-genome summary of PJRB and other strains of *Bacillus* spp. (a) Pan-genome comprising selected strains of *B. safensis*; (b) pan-genome of selected *B. velezensis*; and (c) pan-genome of selected *B. altitudinis* strains. Phylogenetic relationship of *Bacillus* genomes on the basis of pan-genome was depicted in the far left of the diagrams. The matrices represent distribution of genes present or absent in the genome. Each row corresponds to a strain in the panels whereas each column represents an orthologous gene family. Dark blue blocks represent the presence of a gene, while light blue blocks represent the absence of a gene. The pie chart displays distributions of cloud, shell, soft-core, and core genes.

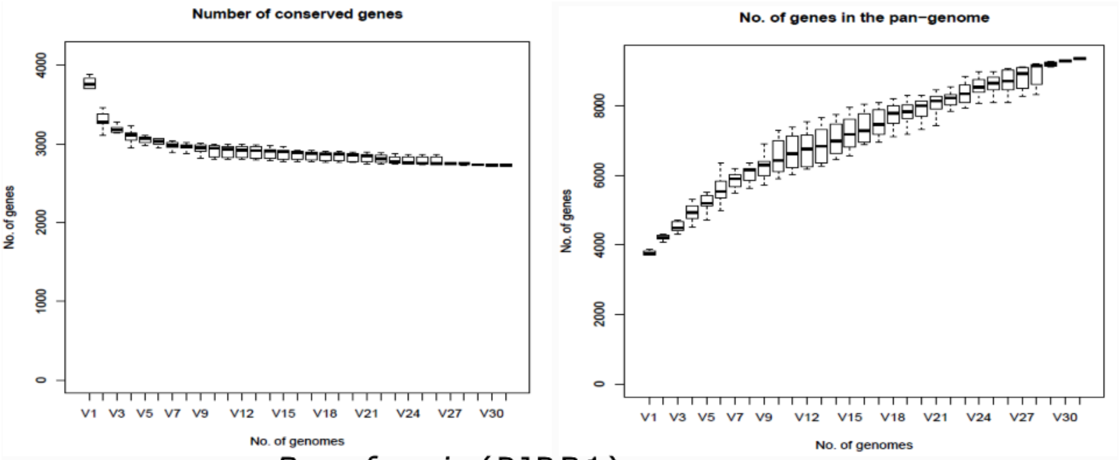

B. safensis (PJRB1) pan genome

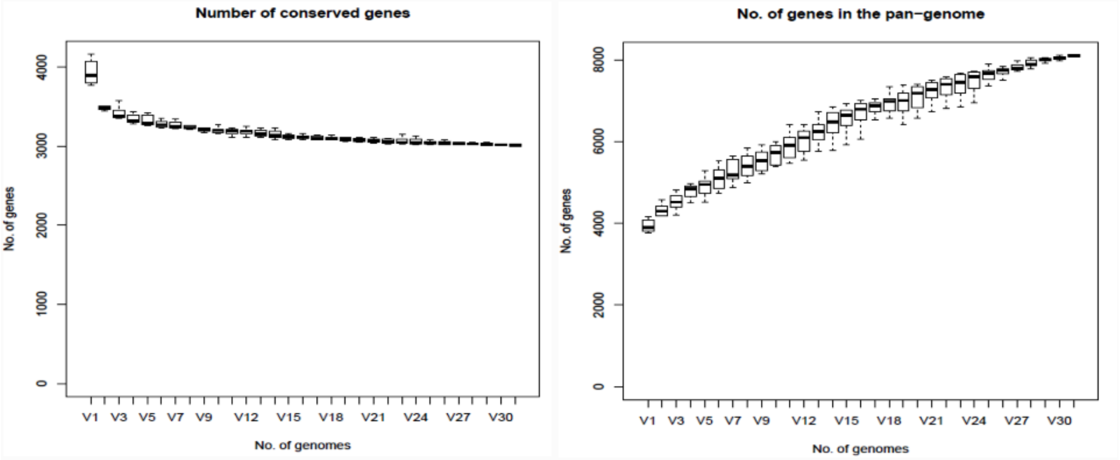

B. velezensis (PJRB2) pan genome

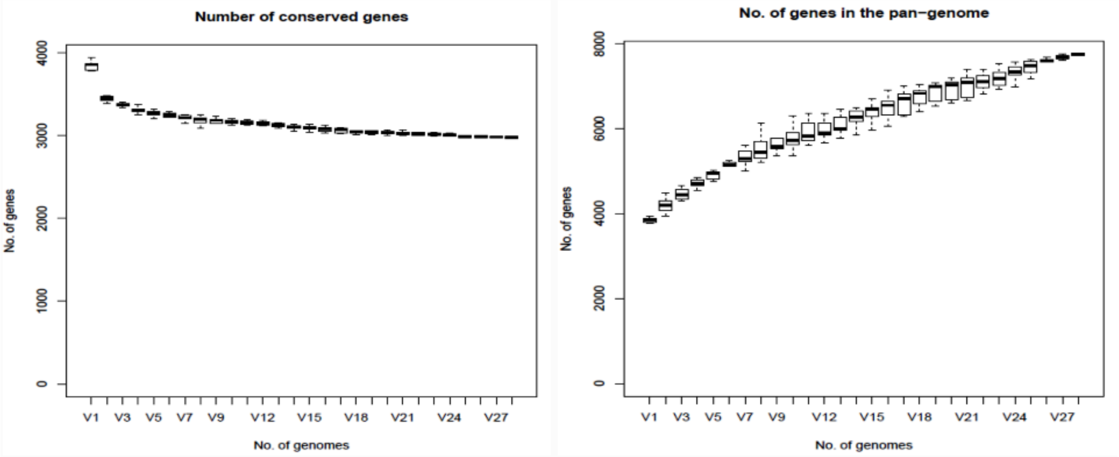

B. altitudinis (PJRB3) pan genome

Fig. S4. The PJRB core genomes and pan-genome as a function of the number of genomes included. The left panel shows saturation of core/conserved genes with the increasing number of genomes whereas the right panel indicates increasing size of the pan-genome as more genomes are included.

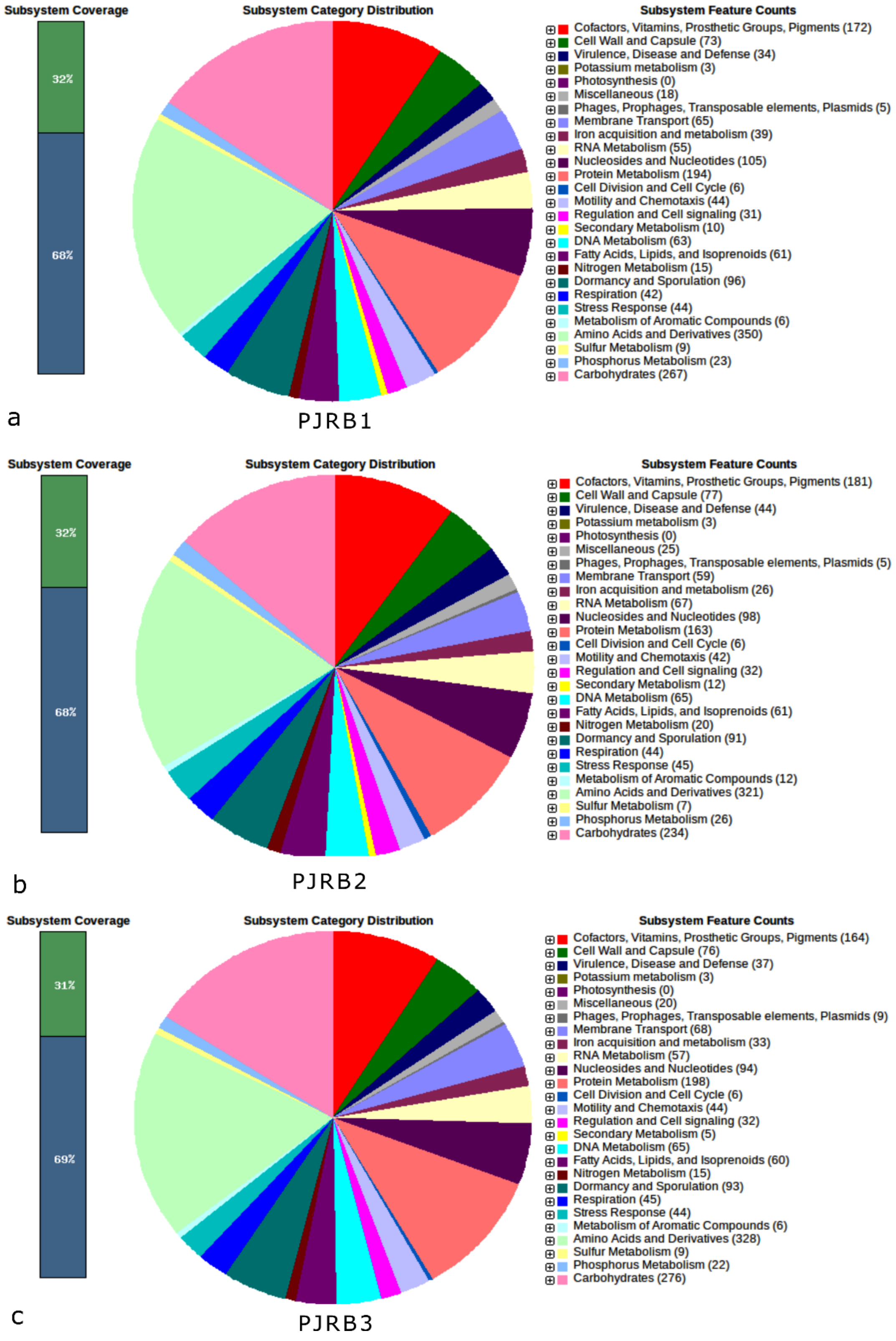

Fig. S5. Subsystem category distribution in the genomes of PJRB strains based on RASTtk annotations. (a) PJRB1, (b) PJRB2, and (c) PJRB3. The subsystem features and coverage of each PJRB genomes are depicted in pie charts. The bars in the left correspond to the percentage of inclusion or exclusion of proteins in the subsystems. The green bars represent included proteins while the blue bar represent proteins that are not included in the subsystems.

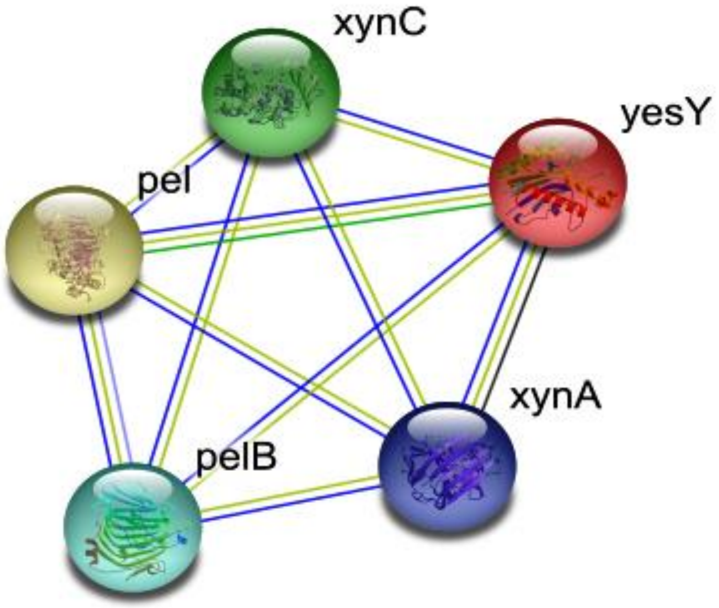

Fig. S6. Protein–protein interaction network of different pectin degrading enzymes in *Bacillus* based on STRING interaction database.
Coloured nodes indicate query proteins and first shell of interactors. The blue lines indicate interactions based on gene co-occurrence, dark green lines based on gene neighbourhood, and light green lines indicate text mining based interactions.

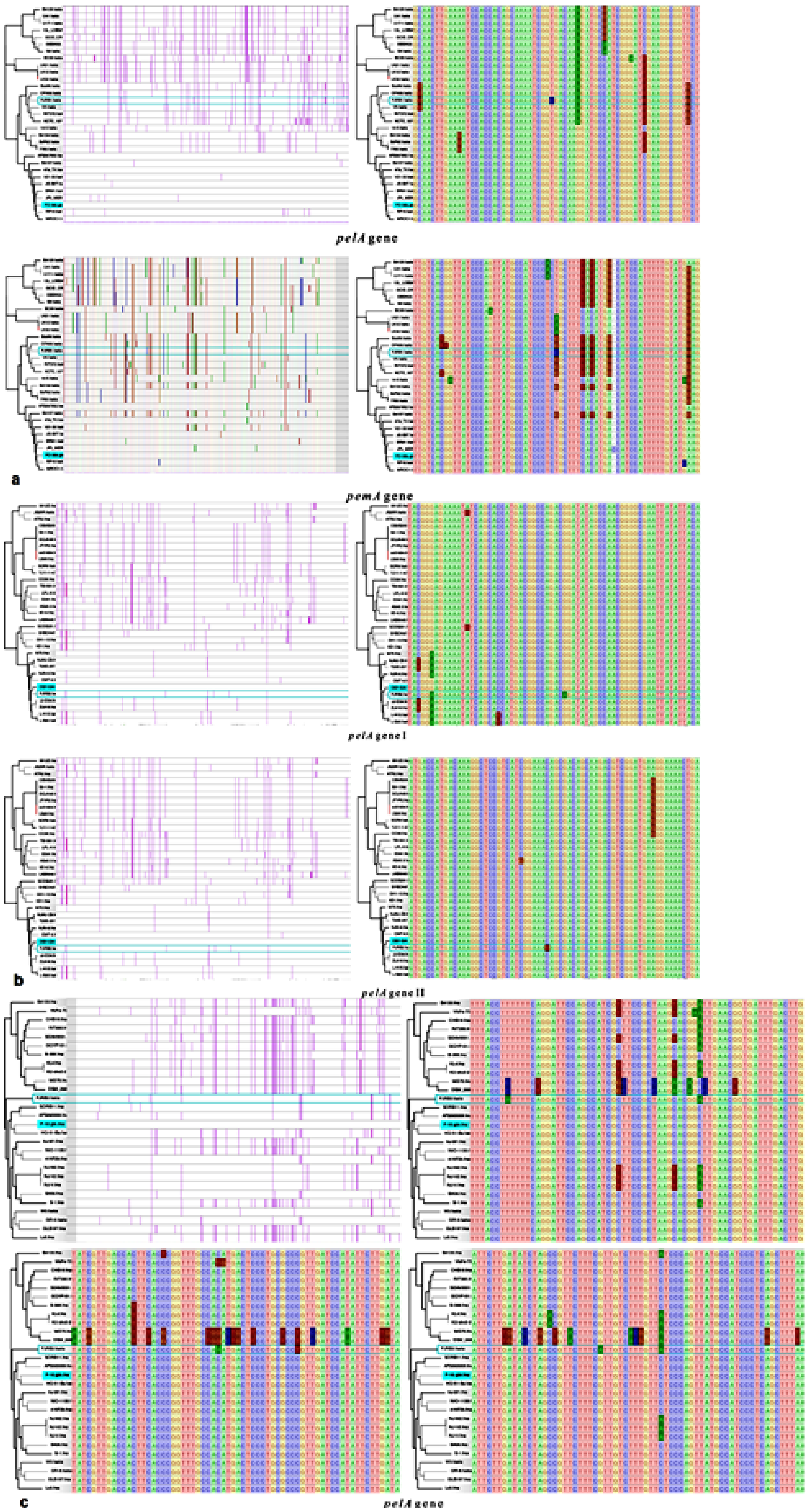

Fig. S7. SNP distribution and base changes in pectin degradation genes of (a) PJRB1, (b) PJRB2, and (c) PJRB3.
